# Single-cell protein secretomic signatures as potential correlates to tumor cell lineage evolution and cell–cell interaction

**DOI:** 10.3389/fonc.2013.00010

**Published:** 2013-02-06

**Authors:** Minsuk Kwak, Luye Mu, Yao Lu, Jonathan J. Chen, Kara Brower, Rong Fan

**Affiliations:** ^1^Department of Biomedical Engineering, Yale UniversityNew Haven, CT, USA; ^2^Department of Electrical Engineering, Yale UniversityNew Haven, CT, USA; ^3^Isoplexis Inc.New Haven, CT, USA; ^4^Yale Comprehensive Cancer CenterNew Haven, CT, USA

**Keywords:** intra-tumor heterogeneity, protein secretion profile, single-cell analysis, immunomonitoring, tumor microenvironment

## Abstract

Secreted proteins including cytokines, chemokines, and growth factors represent important functional regulators mediating a range of cellular behavior and cell–cell paracrine/autocrine signaling, e.g., in the immunological system (Rothenberg, 2007), tumor microenvironment (Hanahan and Weinberg, 2011), or stem cell niche (Gnecchi etal., 2008). Detection of these proteins is of great value not only in basic cell biology but also for diagnosis and therapeutic monitoring of human diseases such as cancer. However, due to co-production of multiple effector proteins from a single cell, referred to as *polyfunctionality*, it is biologically informative to measure a panel of secreted proteins, or secretomic signature, at the level of single cells. Recent evidence further indicates that a genetically identical cell population can give rise to diverse phenotypic differences (Niepel etal., 2009). Non-genetic heterogeneity is also emerging as a potential barrier to accurate monitoring of cellular immunity and effective pharmacological therapies (Cohen etal., 2008; Gascoigne and Taylor, 2008), but can hardly assessed using conventional approaches that do not examine cellular phenotype at the functional level. It is known that cytokines, for example, in the immune system define the effector functions and lineage differentiation of immune cells. In this article, we hypothesize that protein secretion profile may represent a universal measure to identify the definitive correlate in the larger context of cellular functions to dissect cellular heterogeneity and evolutionary lineage relationship in human cancer.

## THE SECRETOMIC PROFILE OF SINGLE T CELLS DEFINES A CORRELATE TO PROTECTIVE IMMUNE RESPONSES

**FIGURE 1:**
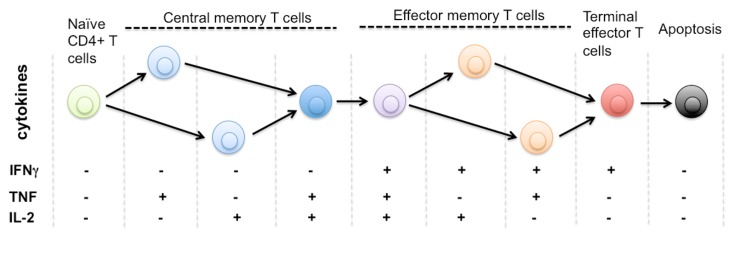
**Protein secretion profile defines phenotype and stage of CD4^+^ T cell differentiation**. It is known that human naïve CD4^+^ T cells differentiate through multiple stages including central memory and effector memory T cells to become terminally differentiated effector T cells that are monofunctional (IFNg^+^), less immunoprotective, and approaching the commitment to apoptosis. The triple positive polyfunctional T cells were found more potent and durable to generate effective immune protection over a prolonged time. Due to the limitation of single-cell cytokine profiling technology, it remains unclear how many effector functions are associated with single T cells. The general trend is polyfunctionality positively correlates to the effectiveness of T cell responses against infection or tissue dysfunction. There is also a diverse range of functional phenotypes defined by distinct cytokine profiles during the process of T cell activation and differentiation. The entire repertoire of heterogeneous T cells collectively defines the quality and protection of T cell-mediated immunity.

To establish our hypothesis and elucidate the strategies, we would like to start with the important discoveries in the field of immunology that have enhanced our understanding of protective immune responses elicited by T cells in response to infection and vaccination. T cells demonstrate diverse and important functional activities in mediating immune response that provide protection against various infections (Precopio et al., 2007; Sallusto and Lanzavecchia, 2009; Bhatia et al., 2012). Upon encountering specific pathogenic antigens that generates polarizing stimulus that induces development of specific phenotype, immune cells are activated and proliferate. After their activation, immune cells differentiated into highly heterogeneous functional lineages and attain a wide variety of effector functions (O’Garra, 1998; Darrah et al., 2007; Precopio et al., 2007; Betts et al., 2006; O’Shea et al., 2008; Seder et al., 2008; Zhu and Paul, 2010; Ma et al., 2011). Effector T cells can regulate and prime their effector mechanisms to clear the infection by producing and secreting diverse cytokines, which play important roles in orchestrating immune responses and controlling pathogenic conditions (Wong and Goeddel, 1986; Harty et al., 2000; Sandberg et al., 2001). T cells develop into highly heterogeneous subpopulations, which can be classified by their differentiation states based on surface marker phenotypes and then by diverse functional profiles (**Figure 1**), as reflected by distinct cytokine production patterns (Sandberg et al., 2001; Appay et al., 2008; Seder et al., 2008; Ma et al., 2011; Han et al., 2012).

The critical issues in developing effective vaccines have been comprehensive characterization of these complex T cell responses (O’Garra, 1998; Darrah et al., 2007; Seder et al., 2008). It is important to identify the correlation of both quality and magnitude of T cell immunity with the protective responses generated following infection or vaccination (De Rosa et al., 2004). Due to increasing phenotypic and functional heterogeneity of effector T cells and the plasticity of T cell differentiation, there have not been clearly defined correlates of immune protection against specific pathogens. Correlate of immune protection is a measurable predictor of an individual’s immunity to a pathogen following infection or vaccination (Zhu and Paul, 2010). Defining correlates of protective T cell immunity has been particularly challenging for immunologists because the degrees of protection does not clearly match with any known T cell phenotypes (O’Shea et al., 2008). Quantification and characterization of these complex and heterogeneous T cell responses have become critical to understand disease pathogenesis and develop preventive or therapeutic vaccines that elicit potent, durable, and specific immune responses (Precopio et al., 2007; Betts et al., 2006; Zhu and Paul, 2010).

The functional profiles of T_H_1 cells (type I helper T cells), one of major functional subsets differentiated from naïve CD4 T cells, demonstrated marked heterogeneity (O’Garra, 1998; Sandberg et al., 2001). Functional analysis of effector T cells using multi-parameter flow cytometry could delineate a number of distinct functional subsets that produce and release different combinations of cytokines within immune response elicited by bacterial infection (De Rosa et al., 2001; Perfetto et al., 2004). The study by De Rosa et al. (2004) measured and characterized the secretion profiles of five cytokines at the single-cell levels using multi-parameter flow cytometry, and discovered that the activated T cells express diverse cytokine profiles. Specific subsets with the ability to produce and secrete multiple cytokines simultaneously conferred more effective and durable protection and other effector functions than the subsets that secreted single cytokines (O’Garra, 1998; Darrah et al., 2007; Appay et al., 2008; Betts et al., 2006; Seder et al., 2008; Han et al., 2012). Frequency of polyfunctional T cells that secreted three distinct cytokines simultaneously and the quality of cytokine secretion best correlated to the degree of protection (De Rosa et al., 2001; Campbell and Polyak, 2007; Polyak, 2011; Marusyk et al., 2012). The induction and maintenance of polyfunctional CD8^+^ T lymphocytes that produce 5+ cytokines contributes to effective anti-viral immune protection (O’Garra, 1998). The immune responses elicited by vaccination that generated optimal protection and resulted in a low level of pathogenic antigens are dominated by multifunctional T cells (Precopio et al., 2007; Zhu and Paul, 2010). Recently a microchip technology allows for simultaneous measurement of up to 12 cytokines to functionally profile antigen-specific CTL (cytotoxic T lymphocytes), which are the main effectors targeting intracellular pathogens (Wong and Goeddel, 1986; Seder et al., 2008; Attig et al., 2009). This device has enabled the detection and characterization of polyfunctional heterogeneity within a phenotypically homogeneous T cell population at single-cell levels. Haining (2012) and Han et al. (2012) used serial microengraving method to design an array of nanowells in which single T cells are isolated and stimulated to cytokine secretion, and characterized the dynamic evolution of cytokine secretion by individual T cells. Recent single immune cell studies also suggest that the ability of effector immune cells to secrete multiple cytokines simultaneously, named polyfunctionality, correlates with protective immune responses (Darrah et al., 2007; Precopio et al., 2007; Betts et al., 2006; O’Shea et al., 2008; Seder et al., 2008; Zhu and Paul, 2010; Ma et al., 2011).

## CELLULAR HETEROGENEITY IN HUMAN CANCER

Almost all solid and metastatic tumors display startling phenotypic and morphologic heterogeneity between and within tumors as well as among different cancer-afflicted individuals (Campbell and Polyak, 2007; Polyak, 2011). Tumor is comprised of highly heterogeneous subpopulation of cells that frequently exhibit substantial variability in virtually all discernible phenotypic features, especially the traits associated with tumorigenesis such as self-renewal capacity, proliferative, invasive, and metastatic potential (Heppner and Miller, 1983; Heppner, 1984; Marusyk and Polyak, 2009; Denysenko et al., 2010; Polyak, 2011; Marusyk et al., 2012). Tumors are not rigid and terminally differentiated cell mixtures, but dynamic organisms which continuously change their properties to adapt to hostile surroundings (Gatenby and Gillies, 2008).

The basic mechanisms by which tumor heterogeneity is evolved and regulated have not been clearly understood and the subject of much discussion (Tu et al., 2002; Michor and Polyak, 2010). Recently, there have been two ideas proposed to elucidate the establishment of tumor heterogeneity (Hanahan and Weinberg, 2011). First, the concept of cancer stem cells (CSCs) postulates that only a small population of cells, or “cancer stem cells,” are responsible for growth, maintenance, and progression of tumors (Reya et al., 2001; Bjerkvig et al., 2005; Ichim and Wells, 2006; Marusyk and Polyak, 2009; Michor and Polyak, 2010). Second, there is the clonal evolution model. The model states that tumor progression is driven as cancer cells over time accumulate highly diverse combinations of genetic and epigenetic alterations (Maley et al., 2006; Marusyk and Polyak, 2009; Sottoriva et al., 2010; Polyak, 2011; Ding et al., 2012). To design an effective and robust personalized therapy that prevents tumor relapse, it is essential to understand the causes and mechanisms of tumor heterogeneity.

Tumor heterogeneity also significantly complicates and impedes investigation and clinical diagnostics of cancer. Because tumor subpopulations exhibit substantial variability in sensitivities to various therapeutic interventions such as chemotherapy, radiation therapy, and immunotherapy, designing effective cancer therapies has posed a major challenge (Håkansson and Tropé, 1974; Hill et al., 1979; Olsson and Ebbesen, 1979; Heppner and Miller, 1983; Schilsky, 1987). One of the major reasons for failure of current cancer therapies is relapse or tumor recurrence after initial remission. Although most cancer cells initially respond to treatment that attempt to selectively kill dividing tumor cells, cancer therapy often fails because there is a small population of cells that re-establish the tumor (Marusyk et al., 2012). Those cells often exhibit potent tumor-initiating capabilities, have intrinsic resistance to treatment, or acquire the mutations that reduce efficacy of treatments (Roche-Lestienne et al., 2003; Mullighan et al., 2008; Ding et al., 2012; Marusyk et al., 2012). In order to stratify patients and predict the therapeutic response, it is required to identify the correlates that can define tumor cell heterogeneity, differentiation stage, lineage relationship, and interactions within a complex microenvironment in the clinical settings.

## SECRETOMIC PROFILES OF SINGLE TUMOR CELLS AS A DEFINITE CORRELATE OF TUMOR HETEROGENEITY AND EVOLUTION

In this article, we would like to introduce a new strategy that may help to assess the extent of tumor heterogeneity, elucidate the fundamental mechanisms of how tumor heterogeneity influence tumor progression and therapeutic responses, and provide valuable insights for designing effective personalized cancer treatments. We hypothesize that a single-cell proteomic secretion profile may be identified as a definite correlate to tumor heterogeneity and evolution. A major challenge in investigating tumor heterogeneity and developing effective diagnostic and therapeutic tools has been the lack of adequate strategies to comprehensively characterize intra-tumor heterogeneity. To fully characterize genetic and phenotypic heterogeneity exhibited within a tumor, the new technologies with the ability to analyze almost every aspect of phenotype at the single-cell level must be developed (Bhatia et al., 2012). Analyzing secretion profiles of soluble mediators such as cytokines and growth factors at single-cell levels is particularly interesting because secretomic profiles of effector T cells can be used to characterize the magnitude and quality of T cell responses and predict a degree of immune protection (Betts et al., 2006; Darrah et al., 2007; Precopio et al., 2007; Seder et al., 2008). Like diverse mixtures of cells constituting tumors, effector T cells exhibit substantial functional and phenotypic heterogeneity, so the similar strategy will be employed to define the extent of tumor heterogeneity and predict tumorigenic potential and drug-resistance. Our preliminary result also suggests that the protein secretion profile evolves as tumor stem cells differentiate.

## EMERGING MICROCHIP TECHNOLOGIES TO ANALYZE SINGLE-CELL PROTEIN SECRETION PROFILES

Defining molecular signatures that indicate the status of human disease or the protective immune response following interventions like vaccines has become one of the central goals in molecular medicine. Characterizing protein secretomic signatures at the single-cell resolution would improve studies of the roles of cellular heterogeneity in pathogenesis, responses to drugs, and cell differentiation (Tay et al., 2010; Agasti et al., 2012). Several new technologies that enabled quantitative single-cell proteomic analysis and characterization of functional and phenotypic heterogeneity shown by diverse cell types have recently been introduced (Fan et al., 2008; Han et al., 2011, 2012; Ma et al., 2011). Many analytical tools have been developed using a wide range of materials and techniques to achieve more efficient isolation of single cells, and multiplexed detection and characterization of secreted proteins (Chin et al., 2004; Rettig and Folch, 2005; Love et al., 2006; Zhu et al., 2009; Han et al., 2011). Recent efforts have reported the development of a novel integrated microfluidic barcode chip platform that enables the rapid, high-content, and multiplexed detection and quantitative assessment of various biomarkers of single cells (Fan et al., 2008; Ma et al., 2011). The integrated blood barcode chip (IBBC) enabled the multiplexed and rapid measurement and quantification of a panel of plasma proteins, including the low abundance cytokines, chemokines implicated in tumor–immune interaction, from a finger prick of human blood (Fan et al., 2008). By integrating microfluidic hydrodynamic principles, the platform enables rapid and effective on-chip blood separation. It employed DNA-encoded antibody library (DEAL) technique, which involves DNA-directed immobilization of antibodies, to create antibody barcode array for *in situ* measurement of plasma proteins (Fan et al., 2008). The single-cell barcode chip (SCBC) has been developed to enable comprehensive characterization of the functional and phenotypic heterogeneity of single immune cells (Ma et al., 2011). The SCBC module consists of a microfluidic system comprised of two polydimethylsiloxane (PDMS) layers and the microscopic slide coated with antibodies (high-density antibody barcode array). The platform has demonstrated multiplexed measurement of a large number of proteins at a single-cell level, and on-chip, rapid, and high-content assessment of protein secretion patterns (Ma et al., 2011). Its capability was validated by detecting multiple cytokine secretions from single macrophages and then polyfunctional profiling of tumor antigen-specific cytotoxic T cells from patients being treated by adoptive T cell transfer therapy (Ma et al., 2011). Varadarajan et al. (2012) reported the design of integrated single-cell analysis to detect and recover antigen-specific CD8^+^ T cells based on their cytokine secretion profiles. Han et al. (2011) introduced an approach based on microengraving that permits quantitative measurements of the rates of cytokine secretion from single immune cells (Olsson and Ebbesen, 1979; Seder et al., 2008). The design minimizes the total number of cells to be interrogated by using a nanowell-array that could retrieve and characterize single CD8^+^ T cells (Love et al., 2006; Han et al., 2011; Varadarajan et al., 2012).

To determine and characterize the protein secretomic profiles of single tumor cells, we have developed and optimized a novel single-cell analysis microchip. This technology will allow for rapid, high-content (more than 1000 single cells), and highly multiplexed measurement of single-cell protein secretion (>14 proteins). The module will be comprised of two major components: ultra-high-density antibody barcode chip and microfluidic single capture platform. We have successfully fabricated a PDMS chip consisting of a sub-nanoliter cell capture microchamber array (unpublished data). The PDMS-based microwell array can rapidly and efficiently capture more than 1000 single cells in a single chip, and the captured cells can be cultured and monitored inside the microchambers that provide physiologically relevant microenvironment. We also aim to employ spectral and spatial multiplexing to significantly increase the number of functional proteins (up to 45 proteins) and single cells (up to 4000 cells) to be analyzed.

To make our platform a more versatile research tool and effective for clinical applications, the high-content and fully automated imaging scheme to image and analyze an entire chip need to be developed. We are in a process of creating novel imaging algorithms with the capacity for detection, counting, and characterization of captured single cells in a rapid and fully automated manner. In order to comprehensively characterize the diverse cellular components, especially highly heterogeneous immune cell compartments, of tumor microenvironment, we are in a process of developing four-color fluorescence imaging to identify phenotypic surface markers of captured single cells for rapid identification of their diverse phenotypes in conjunction of single-cell protein secretion profiling. Integration of these two approaches in a single microchip might provide an effective strategy to define a correlation between distinct cell phenotypes and cytokine secretion, which may lead to improved understanding of the roles of highly heterogeneous cellular components in the tumor microenvironment in promoting tumor development.

## PROTEIN SECRETOMIC PROFILING AS A TOOL TO STUDY THE CYTOKINE NETWORKS MEDIATING COMPLEX TUMOR–MICROENVIRONMENT INTERACTION

Although tumor growth is typically initiated when a single cell acquires genetic abnormalities that confer its proliferative advantages and drive the malignant transformation, tumors do not develop alone, nor are they mere collections of malignant cells with unrestricted proliferation rate (Weiner, 2008; Marusyk et al., 2012; Wu et al., 2012a,b). The decades of research have led to the view that tumor cells actively interact with the tumor microenvironment composed of heterogeneous cell types, and their interplay significantly promotes tumor growth, progression, and metastasis, also drives co-evolution with tumor microenvironment (Mocellin et al., 2001; Dranoff, 2004; Weiner, 2008; Marusyk et al., 2012; Wu et al., 2012a,b). The interplay between these cells comprising the tumor microenvironment are orchestrated by the complex autocrine and paracrine signaling networks, which are mediated by the sets of small soluble proteins such as cytokines, growth factors, and chemokines (Irish et al., 2006; Huang et al., 2007; Raman et al., 2007; Ma et al., 2011). Cytokines are secreted or membrane-bound protein mediators that are involved in diverse biological functions (Dranoff, 2004; Elsawa et al., 2011). When produced in the malignant microenvironment, cytokines and tumor cells form a comprehensive network that have profound influences on tumor growth and progression by modulating the tumor microenvironment (Dranoff, 2004; Sheu et al., 2008; Elsawa et al., 2011). The cytokines such as the tumor-necrosis factor (TNF) are produced by immune cells, and can improve the efficacy of the T cell priming and induce adaptive anti-tumor immunity (Zou, 2005). On the other hand, certain cytokines have been associated with poor patient outcomes, and reported to promote tumor growth and inhibit anti-tumor immune response (Wojtowicz-Praga, 1997; Mocellin et al., 2001; Raman et al., 2007). For example, imbalanced production of interleukin 6 (IL-6), vascular endothelial growth factor (VEGF), or macrophage colony-stimulating factor (M-CSF) inhibit adaptive anti-tumor immunity by suppressing dendritic cell maturation and activating regulatory T cells (T_reg_) to aid tumor cells in evading immune-surveillance (Zou, 2005). Transforming growth factor beta (TGF-β), which is abundantly expressed in many pathological conditions, heavily influence tumor growth and maintenance as the cytokine plays important roles in forming tumor microenvironment, and facilitating angiogenesis (Wojtowicz-Praga, 1997; Zou, 2005; Bierie and Moses, 2006; Sheu et al., 2008).

Targeting and manipulating the cytokine balance have shown the therapeutic efficacy in previous trials (Wojtowicz-Praga, 1997; Zou, 2005; Bierie and Moses, 2006; Sheu et al., 2008; Weidle et al., 2010; Dinarello, 2011). The elucidation of the composition and function of cytokine networks in the tumor microenvironment may identify the targets for potent cancer therapy (Dranoff, 2004; Weiner, 2008). But, a systems-level study, which not just investigates the roles of individual factors, but comprehensively assesses complex signaling networks and recapitulates the dynamics of tumor microenvironment, has yet to be realized (Wu et al., 2012a,b). Despite the importance of characterizing the composition and function of cytokines during tumor development, there have been only a few studies to characterize the complex interplay among different cell types and cytokines within the microenvironment (Egeblad et al., 2008; Shi et al., 2012; Wang et al., 2012; Wu et al., 2012a,b). Shi et al. (2012) developed the SCBC for quantitative and multiplexed assay of intracellular signaling proteins in single tumor cells. The platform can provide a systematic approach to analyze the nature of perturbed signaling transduction networks in the tumor. Wang et al. (2012) utilized the single-cell microchip to assess how cell signaling pathways associated with tumorigenesis are influenced by cell–cell interaction at single-cell levels. To study the tumor microenvironment *in vivo*, Egeblad et al. (2008) developed a multicolor imaging technique to analyze the dynamics and interactions of multiple stromal cell types within the tumor microenvironment via direct observation. Most recently, Yu and his colleagues performed *in silico* stochastic study of glioblastoma multiforme (GMB) microenvironment (Wu et al., 2012a,b). Their model reconstructed the complex cell-to-cell communications in the tumor microenvironment to assess the effects of cytokine-mediated signaling pathways in GMB development. Their model comprises 5 cell types, 15 protein mediators, and 69 signaling pathways, reflecting highly heterogeneous tumor microenvironment (Wu et al., 2012b). This study provides insights into the dynamics of diverse cell populations comprising the tumor microenvironment and the roles of cytokine signaling in the evolution of tumor microenvironment. The cytokine network analysis also identified several key molecules and pathways that play an important role in tumor development and consequently new therapeutic strategies can be designed to target cytokines such as IL-2 and granulocyte-macrophage colony-stimulating factor (GM-CSF), in tumor microenvironment to treat human cancer (Weiner, 2008; Marusyk et al., 2012).

We speculate that the analysis of single tumor cell secretion profiles from a novel clinical microchip will lead to a more complete model that predicts the dynamics of tumor evolution and aids in developing more effective personalized medicine. Each individual tumor cells display unique protein secretion profiles as they secrete unique combinations of cytokines at differing kinetics to regulate widely diverse functions during tumor progression. Significant research efforts have been made recently to develop single-cell proteomics technologies and powerful clinical tools to examine the heterogeneity of tumor microenvironment and complex cytokine-mediated signaling networks, and enable personalized therapy that targets the tumor microenvironment (Irish et al., 2006; Xu et al., 2006; Huang et al., 2007; Ma et al., 2011). Our recently developed single-cell analysis microchip will be employed to experimentally measure the magnitude, quality, and dynamics of cytokine secretion by the cells comprising tumor microenvironment. The single-cell cytokine secretion profile of the tumor will, for the first time, allow reconstruction of a systems-level and large-scale intercellular cytokine signaling network at a single-cell resolution. We also propose to develop new multicolor fluorescence imaging technologies that identify single-cell phenotypic markers and enable rapid molecular phenotyping. By integrating the imaging technologies with single-cell proteomics microchip, we expect to directly assess the behavior of the cells in tumor microenvironment and study how tumor cell cytokine secretion correlates to their phenotypic characteristics and interaction with other cells at the single-cell level. We anticipate that this approach will not only improve cancer diagnosis and stratification but also represents an informative tool to monitor the response of patients, in particular, the one treated by immunotherapy such as cytokine therapeutics, antibody therapy (anti-CTLA4 and anti-PD1), or adoptive T cell therapy that augment the function of anti-tumor immune response in tumor microenvironment to cue cancer (Weiner, 2008).

## IDENTIFICATION OF CANCER STEM CELLS AND LINEAGE DIFFERENTIATION – UNDERSTANDING TUMOR EVOLUTION AND HETEROGENEITY

The CSCs perspective suggests that a small subset of cells with stem cell properties including indefinite proliferative potential is responsible for driving tumor initiation and progression (Reya et al., 2001; Michor and Polyak, 2010). It is one of two major mechanisms that have been proposed to elucidate the origins of tumor heterogeneity. CSCs possess the high self-renewal capacity and unique ability to differentiate, which gives rise to highly heterogeneous cell types that constitute the majority of the tumors, and generates intra-tumor heterogeneity (Hwang-Verslues et al., 2009; Marusyk and Polyak, 2009; Michor and Polyak, 2010). The study by Vermeulen et al. (2008) observed that CSCs from human colon cancer possess multi-lineage differentiation capacity. Recent studies have observed that stem cells are usually preferential targets for mutations that accumulate to cause neoplastic transformation (Bonnet and Dick, 1997; Miyamoto et al., 2000; Betts et al., 2006). CSCs might explain why majority of conventional therapies fail due to tumor relapse after initial remission. It has been suggested that more aggressive cancers that are more likely to relapse contain more CSCs (Al-Hajj et al., 2004; Singh et al., 2004; Bao et al., 2006; Zhou et al., 2009). Many CSCs are relatively more resistant to chemotherapy due to their anti-apoptotic pathways and resistance to oxidative or DNA damage (Reya et al., 2001; Li et al., 2006; Diehn et al., 2009).

**FIGURE 2:**
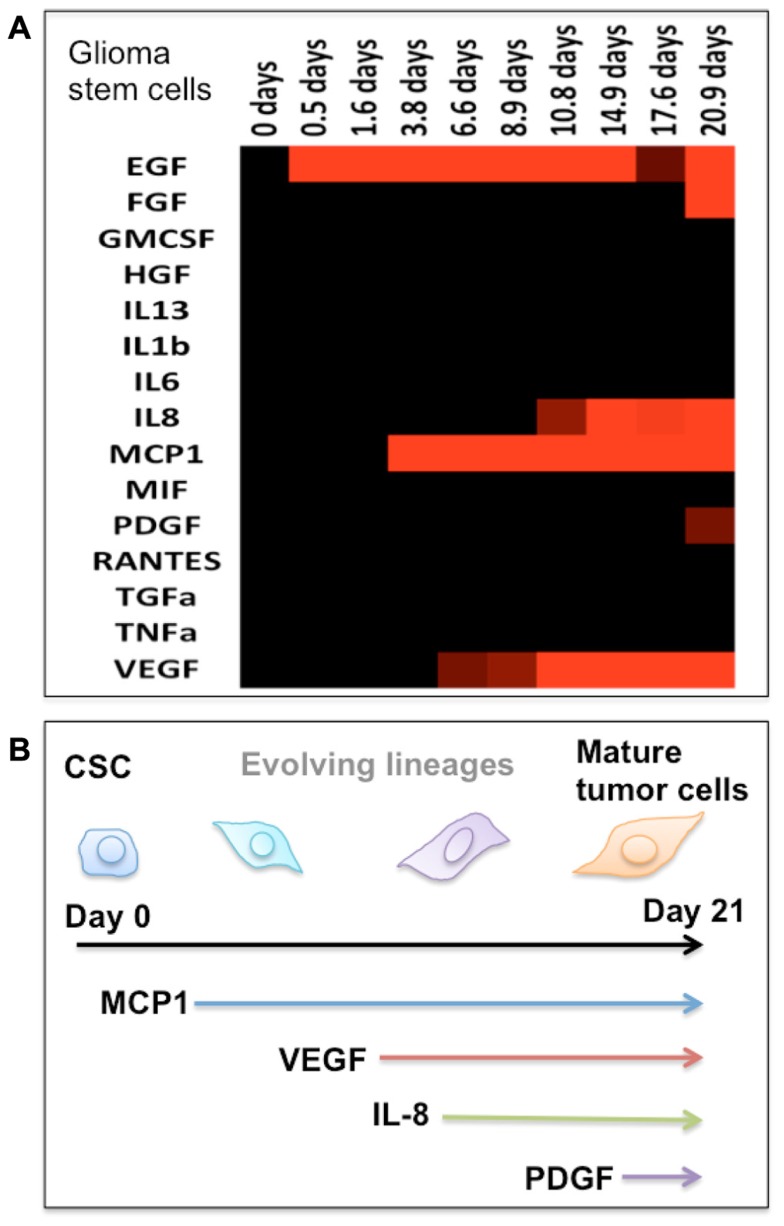
**Protein secretion profile correlates to the stage of cancer stem cell differentiation**. **(A)** Heat map showing the protein secretion profiles measured at different times during the differentiation of human glioma stem cells *in vitro*. **(B)** Schematic depiction of the stage of glioma stem cell differentiation as identified by emergence of cytokine secretion. In our protein assay panel, glioma stem cells appear to be relatively “quiescent.” During the differentiation process, these cells begin to produce a number of functional proteins and gradually become the phenotype of mature tumor cells.

However, the definitive cellular or molecular biomarkers that identify tumor-initiating cells have not yet determined. The study by Hwang-Verslues et al. (2009) identified different subpopulations of cells displaying distinct tumorigenic abilities within the breast cancer cell line. The discoveries suggest that there are multiple lineages of CSCs, which can subsequently be differentiated into more diverse cells. The CSC perspective views the tumors as hierarchical organization composed of multiple lineages of differentiated cells with distinct phenotypes. The analysis of single-cell secretomic profiles shows that while every single cell exhibits distinct secretomic profiles, there are groups of single cells with comparable secretomic profiles. Based on single-cell secretomic profiles, the entire tumor cell population may be compartmentalized into multiple clusters, each of which is a group of cells that have similar or related cytokine secretion patterns. The multiple groups of single cells classified based on the secretion profile may represent distinct lineages originated from the differentiation and evolution of CSCs. Our study has shown marked change of protein secretion profiles from human brain tumor cells undergoing differentiation to mature tumor cells, suggesting the possibility of using secretomic signatures to define tumor cell differentiation and heterogeneity (**Figure 2**).

The mechanisms by which CSCs acquire their tumorigenic and metastatic abilities to promote tumor growth, metastasis, and resistance to therapy have not been fully understood. As normal stem cells are influenced by their “niche,” CSCs are regulated by, and in turn regulate, the extrinsic signals generated within the tumor microenvironment (Karnoub et al., 2007; Weiner, 2008; Korkaya et al., 2011). Heterogeneous cell types that constitute the tumor microenvironment secrete the pro-inflammatory cytokines such as IL-6 or IL-8 that increase tumorigenic potential and promote therapeutic-resistance (Scheller et al., 2006; Levina et al., 2008; Liu et al., 2011). In turn, tumorigenic cells also produce and secrete various factors to enhance their survival and proliferation. Recent studies have found that the capabilities of CSCs to sustain tumor growth and promote resistance to various therapies are associated with their high ability to produce soluble mediating factors such as cytokines and growth factors (Todaro et al., 2007; Weiner, 2008; Iliopoulos et al., 2009; Tang et al., 2012). The study discovered that the levels of numerous cytokines, growth factors, and chemokines were two- to threefolds higher in isolated CSC-derived tumors than parental tumor cells (Levina et al., 2008; Tang et al., 2012). The production of IL-4 by colon CSCs contributes to higher therapeutic-resistance as IL-4 promotes the expression of anti-apoptotic genes and upregulates resistance to apoptosis of CSCs (Todaro et al., 2007; Iliopoulos et al., 2009). These studies suggest that the greater ability to produce multiple cytokines has been correlated to tumorigenic and metastatic potential. From the single-cell secretion profiles, we can identify the groups of tumor cells characterized by the significant secretion of multiple, specific cytokines. These groups may elicit greater tumorigenic potentials and promote the evolution of more aggressive and invasive cancer phenotypes. Because our single-cell analysis microchip allows comprehensive characterization of phenotypes of captured single cells, including their surface phenotypes, motility, and viability, we hope to determine the correlation between specific cytokine secretion profiles of individual cells and their tumorigenic potentials and differentiation stages. The cytokine secretion profiles of single tumor cells can be used to characterize a tumor hierarchy and serve as biomarkers for tumor-initiating cells or different lineages with varying tumorigenicity and treatment-resistance.

## OUTLOOK, CLINICAL APPLICATION, AND UTILITY

The effective targeting of cancerous cells with greater tumorigenic potential and intrinsic drug-resistance can prevent cancer relapse or persistent growth, and when combined with conventional therapy that kills the rapidly dividing cells, it can potentially cure cancer (Vermeulen et al., 2008; Zhou et al., 2009; Chen et al., 2010; Michor and Polyak, 2010). Our single cell-based cytokine secretion analysis would provide framework and new insight for designing effective therapeutic strategies by dissecting hierarchical organization of tumor microenvironment in hope to identify the specific cell subsets with higher tumorigenic and metastatic potential, and resistance to treatment. Single-cell secretomic profiling could become a new means for quantitative characterization of the extent of tumor heterogeneity, with which oncologists can diagnose the stage of cancer and likelihood of development of metastatic cancer for individual patients, leading to personalized medicine and treatments. Because distinct cytokine secretion patterns are associated with distinct differentiation lineages, secretomic profiling may aid in understanding of CSC differentiation and tumor evolution.

One of the major challenges in designing effective personalized cancer therapeutics and early diagnosis has been the lack of adequate technologies to comprehensively characterize inter- and intra-tumor heterogeneity in the clinical settings. Single-cell analysis of cytokine profiles are possible correlates to evaluate whether there is a high degree of intra-tumor heterogeneity of cancer phenotypes, and provide valuable insights into the origins of tumor heterogeneity, the mechanisms of the complex signaling networks that mediate the characteristics of individual tumor cells, and the extent of tumor differentiation and evolution, that has the potential to enable the development of more effective personalized medicines for human cancers.

## Conflict of Interest Statement

The authors declare that the research was conducted in the absence of any commercial or financial relationships that could be construed as a potential conflict of interest.
